# Correlation of supplement folic acid based on MTHFR and MTRR gene polymorphisms with preeclampsia in Chinese population: an retrospective cohort study

**DOI:** 10.3389/fnut.2025.1729915

**Published:** 2025-12-15

**Authors:** Xuanjun Xiong, Lin Xiao, Xiaoqin Xin, Jungao Huang

**Affiliations:** 1 Ganzhou Maternal and Child Health Hospital, Ganzhou, Jiangxi, China; 2 Department of Clinical Laboratory, Ganzhou People’s Hospital, Ganzhou, Jiangxi, China

**Keywords:** preeclampsia, folic acid, genetic factor, correlation, Chinese population

## Abstract

**Background and objectives:**

The relationship between folic acid supplementation and preeclampsia remains a topic of ongoing debate. We aimed to investigate the correlation between folic acid and preeclampsia from a genetic perspective.

**Methods:**

This retrospective cohort included 377 Chinese participants. We evaluated the risks of eclampsia, including placental growth factor (PIGF) indicators, and the genotypes of folate metabolism markers (MTHFR C677T, MTHFR A1298C, and MTRR A66G). Based on these polymorphisms, participants were categorized into high-risk and relatively low-risk groups, with the high-risk group receiving folic acid supplementation. Multivariate linear and logistic regressions were utilized to calculate the *β*-coefficients, odds ratios (ORs), and 95% confidence intervals (CIs).

**Results:**

PlGF levels were negatively associated with preeclampsia, with each one-unit increase in PlGF corresponding to a 15% reduction in preeclampsia risk (OR = 0.85, 95% CI: 0.8–0.9), a finding that remained stable across various models. Interestingly, a significant difference was found in PIGF levels between the high-risk group for folic acid and the low-risk group (*p* = 0.01). Moreover, there was a significantly positive correlation (*β* = 11.11, 95% CI: 1.68–20.53) and the association persisted across different models, which indicated the high-risk group showed an increase in PIGF concentration when supplementing with folic acid.

**Conclusion:**

To our knowledge, limited studies have examined this association at the genetic level in the Chinese population. Our findings suggest that folic acid may mitigate the risk of preeclampsia through its effects on PIGF levels. Potentially, folic acid supplementation could serve as a preventative strategy against preeclampsia.

## Introduction

Preeclampsia is a multifaceted disorder occurring exclusively during pregnancy and is a leading cause of maternal and neonatal morbidity and mortality worldwide (1). It affects approximately 3% to 8% of all pregnancies, contributing to an estimated 10% to 15% of maternal fatalities (2, 3). The pathophysiology of preeclampsia remains poorly understood, as its manifestations are diverse and complex. Currently, there are no effective treatments to halt the progression of the disease, making expedited delivery of both the fetus and placenta the only viable intervention (4). Early preeclampsia is often linked to placental dysfunction, with placental growth factor (PLGF) implicated in endothelial dysfunction in affected individuals. PLGF has been successfully utilized as a biomarker for diagnosing and predicting preeclampsia (5, 6). There are already methods for automatically measuring PIGF that have been used to assess the risk of preeclampsia (7).

Folic acid is an essential cofactor in one-carbon metabolism, critical for DNA methylation, repair, cell division, and embryonic development. Its demand increases substantially during pregnancy due to the rapid proliferation of placenta and fetal cells (8). Numerous studies have investigated the relationship between folic acid supplementation and preeclampsia; however, the results have been inconsistent. Some research indicates that folic acid is associated with a decreased risk of preeclampsia (8, 9). In contrast, other studies have found no significant correlation between folic acid supplementation and preeclampsia risk (10, 11). Notably, studies have demonstrated that individualized folic acid supplementation, tailored according to the polymorphisms of methylenetetrahydrofolate reductase (MTHFR) and methionine synthase reductase (MTRR), can significantly reduce pregnancy complications (12, 13). MTHFR plays a critical role in the folate metabolism pathway by catalyzing the conversion of 5,10-methylenetetrahydrofolate to 5-methyltetrahydrofolate, a crucial step in the remethylation of homocysteine to methionine. MTRR is also important for the metabolism of homocysteine and the synthesis of methionine. It functions as a reductase that regenerates methionine synthase, allowing for the conversion of homocysteine to methionine. This process is essential for numerous metabolic activities, including the synthesis of S-adenosylmethionine (SAM), a key methyl donor in various biochemical reactions. Variations in the MTHFR and MTRR genes can significantly impact folate metabolism (12). However, many previous studies have failed to consider the influence of genetic factors on the relationship between folic acid and preeclampsia, particularly individual genetic polymorphisms.

In light of this oversight, we aimed to investigate the correlation between folic acid supplementation and the risk of preeclampsia, specifically focusing on the polymorphisms of the MTHFR and MTRR genes. Our goal is attempting to contribute to the development of more effective, personalized strategies for folic acid supplementation during pregnancy.

## Participants and methods

Before the analysis, we used the G * Power 3.1 tool to ensure the adequacy of the sample size. This study involved a total of 377 participants from our hospital in Ganzhou, China, and recruited between October 2024 and September 2025, with ages ranging from 19 to 41 years. The mean gestational age was 12^+3^ weeks. Exclusion criteria included: (1) patients with diabetes, chronic hypertension, or systemic lupus erythematosus; (2) smokers; (3) pregnant women with a history of preeclampsia or a family history of the condition; and (4) participants missing key covariates. Inclusion criteria required that participants underwent both folate metabolism gene testing and preeclampsia risk assessment. Based on the results of the folic acid gene test, subjects were divided into a high-risk group and a relatively low-risk group. Participants with mutations in both MTHFR and MTRR genes were classified as high-risk. Specifically, individuals carrying the variant alleles of MTHFR and MTRR are detailed in Table 1, while those with at least one wild-type allele for either gene were classified as relatively low-risk. The high-risk group received folic acid supplementation, while the low-risk group in the control group did not receive folic acid supplementation in their daily diet. Written informed consent was obtained from all participants. The primary data, including clinical records and laboratory data, were retrieved from the hospital’s HIS (Hospital Information System) and stored and managed by the hospital’s information management department. All retrospective data were collected from the Ganzhou Maternal and Child Health Hospital, and we received approval from the ethics committee for its use.

**Table 1:** Genotypes and loci associated with high risk for folate and folic acid supplementation in clinical settings.

Genotype			Folic acid supplementation		
MTHFR (c.1298A > C)	MTHFR (c.677 C > T)	MTRR (c.66 A > G)	3 months before conception	Early pregnancy (0–12 weeks)	Late pregnancy (13–40 weeks)
C/C	C/C	A/G	800 μg/day	800 μg/day	800 μg/day
C/C	C/C	G/G	Individuals with MTRR c.66 A/G or G/G also need to supplement vitamins B12 2.4 μg/d		
C/C	C/T	A/G			
C/C	C/T	G/G			
C/C	T/T	A/G			
C/C	T/T	G/G			
A/C	C/C	A/G			
A/C	C/C	G/G			
A/C	C/T	A/G			
A/C	C/T	G/G			
A/C	T/T	A/G			
A/C	T/T	G/G			
A/A	C/T	A/G			
A/A	C/T	G/G			
A/A	T/T	A/G			
A/A	T/T	G/G			

In this study, we categorized race/ethnicity as Han and Other. Crown-rump length (CRL), number of fetuses, and gestational age were measured via ultrasound. Pregnancy history and past medical history were collected through consultations with a qualified physician. Weight, height, and mean arterial pressure (MAP) were assessed by professional nurses. The risk of folic acid metabolism was determined based on polymorphisms in the MTHFR and MTRR genes. DNA was extracted using standard methods, and in our laboratory, we employed the PCR-melting curve method with commercial reagents to detect genotypes for MTHFR C677T, MTHFR A1298C, and MTRR A66G (Tianlong, China). The preeclampsia risk assessment model used in this study has been widely implemented in our hospital for many years. Additionally, placental growth factor (PlGF) concentration was measured using an automatic chemiluminescence immunoassay analyzer (Perkin Elmer, Finland).

## Statistical analysis

Statistical analyses were conducted using R software (http://www.R-project.org, The R Foundation) and Free Statistics software version 1.4. Categorical variables are presented as counts (*n*) and percentages (%), while continuous variables are expressed as means and standard deviations. The statistical methods used in the analyses included the chi-square test, two-tailed Student *t*-test, and both logistic and linear regression.

In Model 1, no variables were accounted for during the univariate analysis. Model 2 included age, race, height, and weight in the analysis. Based on Model 2, all covariates listed in Table 2 were adjusted for in Model 3. Odds ratios (ORs) and 95% confidence intervals (CIs) were calculated in the multivariate logistic analysis. For the multivariate linear regression models, *β*-coefficients and 95% CIs were determined. Varianceinflation factors (VIF) were calculated to assess multicollinearity, and no VIF values exceeding 10 were found. During the process of analysis, Shapiro–Wilk test and visually inspected Q-Q plots were employed to assess the normality of residuals. Potential interaction was evaluated using the likelihood ratio test and no substantial interactions that would necessitate further analysis. A two-sided *p*-value of less than 0.05 was considered statistically significant.

**Table 2:** The characteristics of the study population by quartiles of PlGF concentration.

Variables	Total (*n* = 377)	Q1 (*n* = 94)	Q2 (*n* = 94)	Q3 (*n* = 94)	Q4 (*n* = 95)	*p* value
Age (years), Mean ± SD	29.3 ± 4.1	28.8 ± 4.3	28.8 ± 4.5	29.4 ± 3.9	30.1 ± 3.6	0.099
Race, *n* (%)						0.245
Han	375 (99.5)	94 (100)	94 (100)	94 (100)	93 (97.9)	
Other	2 (0.5)	0 (0)	0 (0)	0 (0)	2 (2.1)	
CRL (mm), Mean ± SD	58.4 ± 5.8	57.3 ± 5.2	57.0 ± 5.7	57.9 ± 5.0	61.3 ± 6.2	< 0.001
MAP (mmHg), Mean ± SD	79.8 ± 7.2	81.2 ± 6.6	80.1 ± 7.1	79.9 ± 7.6	78.2 ± 7.1	0.033
Gestational weeks, Mean ± SD	12.4 ± 0.5	12.3 ± 0.4	12.2 ± 0.4	12.3 ± 0.4	12.6 ± 0.5	< 0.001
Height (cm), Mean ± SD	158.9 ± 5.4	159.6 ± 4.9	159.5 ± 5.7	158.7 ± 5.4	158.0 ± 5.6	0.149
Number of fetuses, *n* (%)						1
Single	371 (98.4)	93 (98.9)	92 (97.9)	93 (98.9)	93 (97.9)	
Two	6 (1.6)	1 (1.1)	2 (2.1)	1 (1.1)	2 (2.1)	
Weight (kg), Mean ± SD	55.4 ± 9.2	55.5 ± 9.7	55.7 ± 9.3	55.8 ± 8.7	54.6 ± 9.0	0.788
Pre-delivery age, Mean ± SD	30.3 ± 4.1	29.8 ± 4.3	29.8 ± 4.5	30.4 ± 3.9	31.1 ± 3.6	0.088
Pregnancy history (<24 week), *n* (%)						0.134
No	315 (83.6)	81 (86.2)	84 (89.4)	73 (77.7)	77 (81.1)	
Yes	62 (16.4)	13 (13.8)	10 (10.6)	21 (22.3)	18 (18.9)	
Risk of folic acid, *n* (%)						0.009
Low	347 (92.0)	90 (95.7)	90 (95.7)	87 (92.6)	80 (84.2)	
High	30 (8.0)	4 (4.3)	4 (4.3)	7 (7.4)	15 (15.8)	
Risk of eclampsia, *n* (%)						<0.001
Low	341 (90.5)	63 (67)	92 (97.9)	93 (98.9)	93 (97.9)	
High	36 (9.5)	31 (33)	2 (2.1)	1 (1.1)	2 (2.1)	

Q, quartiles; CRL, crown-rump length; MAP, mean arterial pressure.

## Results

The baseline characteristics of the study population are summarized in Table 2 according to quartiles of placental growth factor (PlGF) concentration. A total of 377 participants were included in the final analysis, with a mean age of 29.3 ± 4.1 years, predominantly of Han ethnicity (99.50%). The risks associated with high folic acid metabolism and preeclampsia were found to be 8.0% and 9.5%, respectively. At baseline, characteristics varied between PlGF quartiles for crown-rump length (CRL), mean arterial pressure (MAP), gestational weeks, folic acid risk, and eclampsia risk.

As shown in Table 3, PlGF concentration exhibited a significant negative correlation with the risk of preeclampsia. For every one-unit increase in PlGF, the risk of preeclampsia decreased by 15% (OR = 0.85, 95% CI: 0.80–0.90). This association remained stable across different models (Crude Model, Model 1, and Model 2). To further investigate this relationship, we divided PlGF into four equal parts and found that an increase in PlGF concentration served as a protective factor, consistently reducing the risk of preeclampsia across all cases. Specifically, the risk of preeclampsia was reduced by approximately 97% (OR = 0.03, 95% CI: 0.01–0.17) in the fully adjusted model when comparing the highest quartile (Q4) to the lowest quartile (Q1). The trend test also indicated a significant association (*p* for trend <0.001).

**Table 3:** Odds ratios (95% confidence intervals) of risk of eclampsia across quartiles of PlGF concentration.

Item	Event (%)	Crude model		Model 1		Model 2	
		OR (95% CI)	*p* value	OR (95% CI)	*p* value	OR (95% CI)	*p* value
PlGF (pg/ml)	36 (9.5)	0.87 (0.83 ~ 0.91)	<0.001	0.87 (0.83 ~ 0.91)	<0.001	0.85 (0.8 ~ 0.9)	<0.001
Q1 (<7.57)	31 (33)	1 (Ref)		1 (Ref)		1 (Ref)	
Q2 (7.57–30.10)	2 (2.1)	0.04 (0.01–0.19)	<0.001	0.04 (0.01–0.19)	<0.001	0.03 (0.01–0.16)	<0.001
Q3 (30.10–57.10)	1 (1.1)	0.02 (0–0.16)	<0.001	0.02 (0–0.15)	<0.001	0.01 (0–0.1)	<0.001
Q4 (>57.10)	2 (2.1)	0.04 (0.01–0.19)	<0.001	0.04 (0.01–0.18)	<0.001	0.03 (0.01–0.17)	<0.001
*P* for trend		0.18 (0.1–0.34)	<0.001	0.18 (0.1–0.33)	<0.001	0.15 (0.08–0.31)	<0.001

PlGF, placental growth factor; Ref, reference; OR, odds ratios; CI, confidence intervals.

Crude model adjusted for none.

Model 1 adjusted for age, race, height, and weight.

Model 2 adjusted for all covariates listed in Table 2.

**Figure 1:**
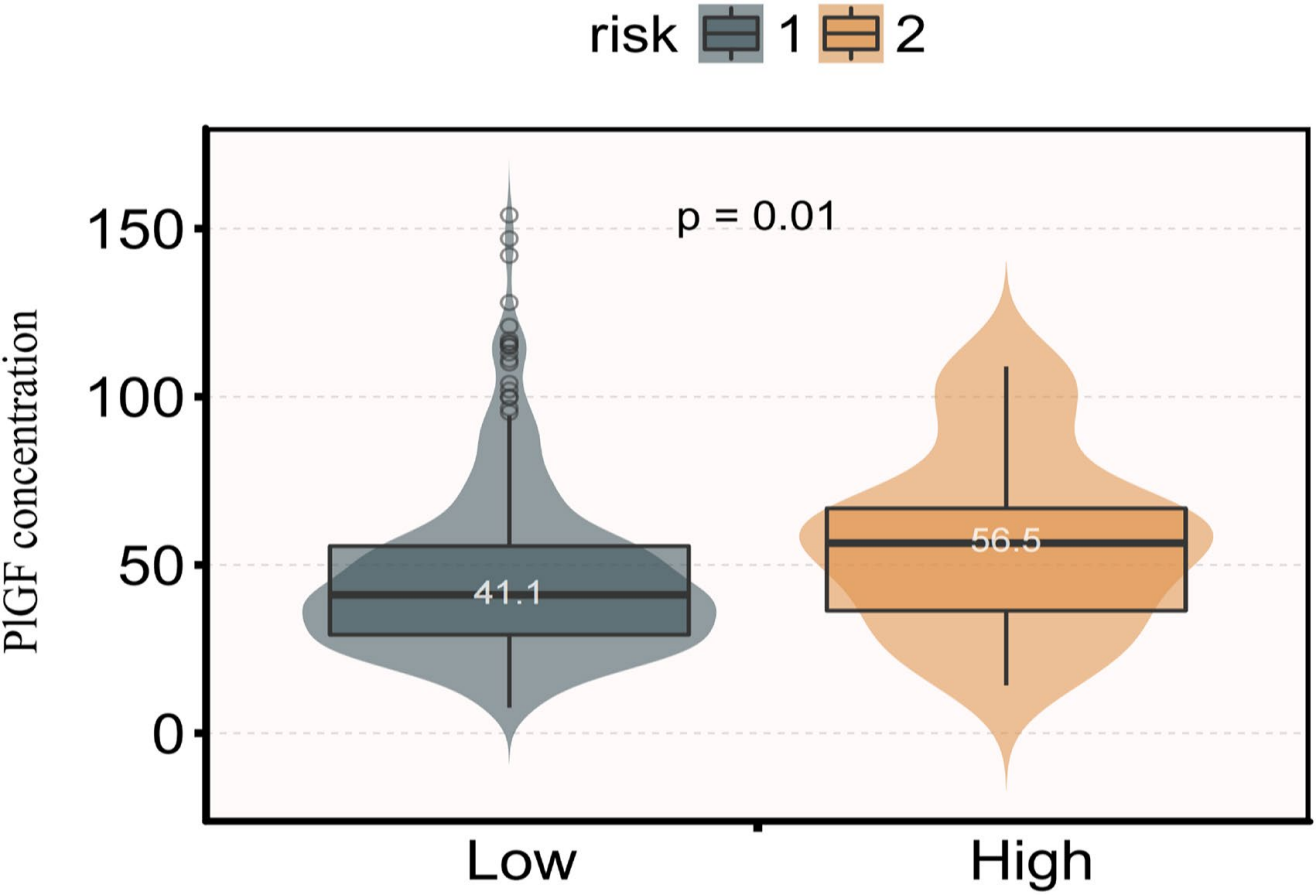
Distribution of PlGF concentration in participants by risk of folic acid. PlGF, placental growth factor; risk 1, relatively low-risk group; risk 2, relatively high-risk group.

Interestingly, we observed a significant difference in PlGF levels between the high-risk and low-risk groups for folic acid metabolism (*p* = 0.01). The PlGF concentrations for the two groups were 56.5 pg/mL and 41.1 pg/mL, respectively (Figure 1). Furthermore, a multivariate linear regression model was employed to explore the relationship between folic acid and PlGF levels. In Model 2, the PlGF level was significantly higher in the high-risk group compared to the low-risk group, showing a positive correlation (*β* = 11.11, 95% CI: 1.68–20.53). This association was consistent across the Crude Model, Model 1, and Model 2 (see Table 4).

**Table 4:** Associations between PlGF concentration and folic acid supplement.

Risk of folic acid metabolism	*n*	Crude model		Model 1		Model 2	
		*β*-coefficient (95% CI)	*p* value	*β*-coefficient (95% CI)	*p* value	*β*-coefficient (95% CI)	*p* value
Low	347	0 (Ref)		0 (Ref)		0 (Ref)	
High	30	11.14 (1.82 ~ 20.46)	0.02	11.33 (2.01 ~ 20.66)	0.018	11.11 (1.68 ~ 20.53)	0.021

Ref, reference; CI, confidence intervals.

Crude model adjusted for none.

Model 1 adjusted for age, race, height, and weight.

Model 2 adjusted for all covariates listed in Table 2.

**Figure 2:**
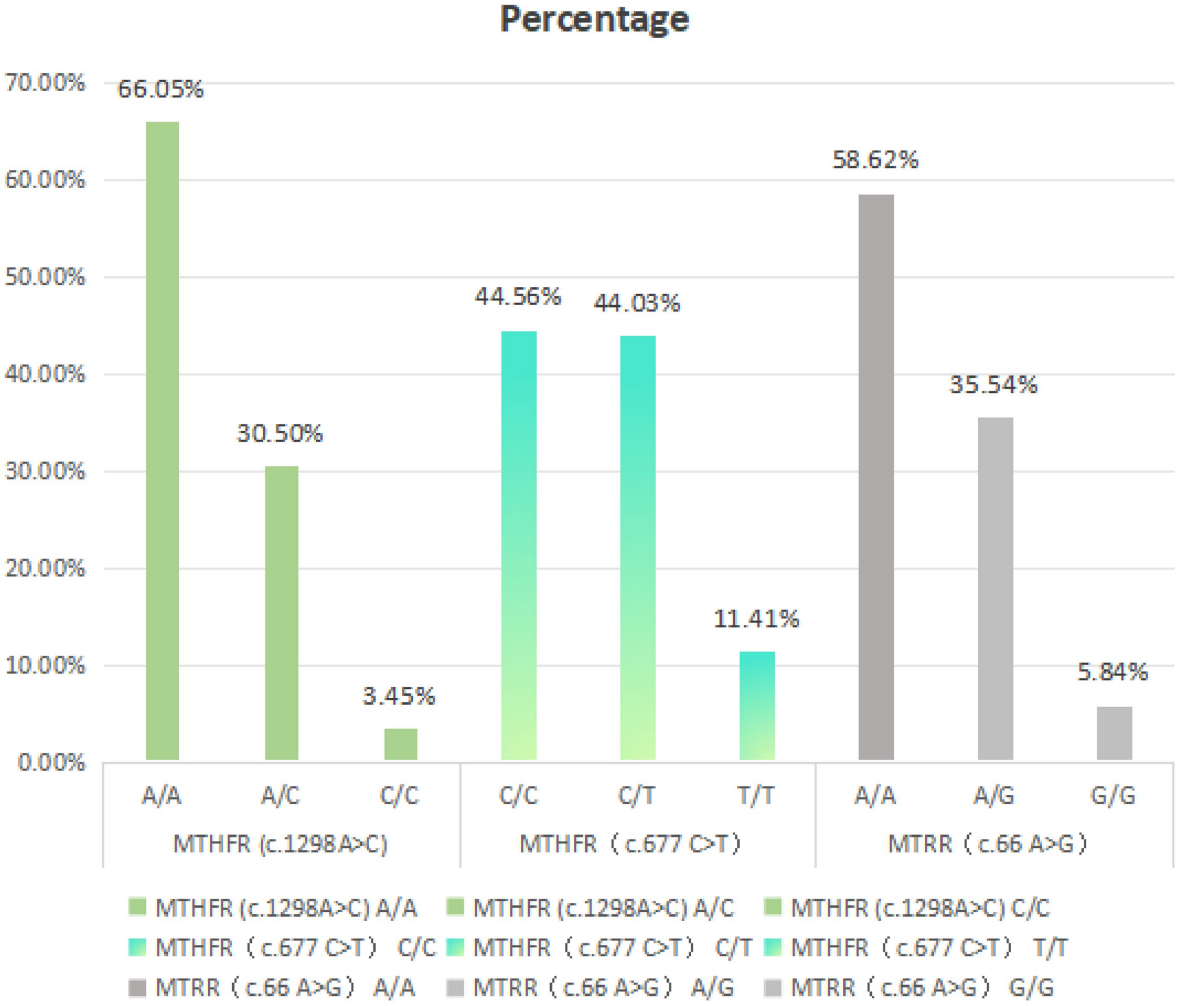
Distribution of alleles for polymorphisms in MTHFR and MTRR genes.

Table 1 presents the various high-risk genotypes associated with folic acid and suggests appropriate folic acid supplementation at different clinical time points. We further analyzed the distribution of each genotype related to folate metabolism within the population. The results indicated that MTHFR (A1298A) and MTRR (A66A) comprised the higher proportions, accounting for 66.05% and 58.62%, respectively. Among the mutant alleles, MTHFR (C677T) and MTRR (A66G) also represented significant proportions, comprising 44.03% and 35.54%, respectively (Figure 2).

## Discussion

In the present study, we investigated the correlation between folic acid supplementation, as determined by MTHFR and MTRR gene polymorphisms, and preeclampsia. Our results confirmed the relationship between placental growth factor (PlGF) levels and the incidence of preeclampsia (14, 15). Specifically, we found that for every one-unit increase in PlGF, the risk of preeclampsia decreased by 15% (OR = 0.85, 95% CI: 0.80–0.90).

PlGF is located in the q24-q31 region of chromosome 14 and is a secretory dimeric glycoprotein with a molecular weight of 45–50 kDa, predominantly expressed in placental trophoblast cells during pregnancy. Congestion of the placental villi exerts pressure on the syncytiotrophoblast cells, impeding oxygen supply. Consequently, PlGF levels significantly decrease, which can lead to impaired placental development and function. This dysfunction makes it challenging for the placenta to provide adequate nutrients and oxygen to the fetus, potentially triggering preeclampsia (6). In fact, serum PlGF concentrations in pregnant women with preeclampsia have been shown to decrease significantly (16).

Previous studies have demonstrated a link between folate deficiency and preeclampsia. A cohort study by Wang et al. (17), which included 10,041 pregnant women, found that folic acid supplementation during pregnancy, along with increased dietary folate intake, could reduce the risk of preeclampsia. Additional research has indicated that folic acid intake during pregnancy can lower the risk of preeclampsia, particularly among women already at heightened risk (18, 19). Supporters of folic acid supplementation argue that this approach is biologically plausible, as patients with eclampsia tend to have significantly elevated homocysteine levels. Supplementing with folic acid can reduce these levels, thereby potentially lowering the risk of eclampsia during pregnancy (17).

However, some studies have reported conflicting conclusions. A recent meta-analysis by Cui et al. suggested that folic acid supplementation alone was not associated with a reduced risk of preeclampsia (8). It is possible that previous research did not adequately consider individual heterogeneity, as the required amount of folic acid can vary significantly among individuals, contributing to discrepancies in findings. Moreover, a recent study indicated that personalized folic acid supplementation based on polymorphisms in the MTHFR and MTRR genes could reduce the risk of gestational diabetes (20). Another study established that the MTHFR C677T polymorphism was associated with an increased risk of Down syndrome in mothers, while the MTHFR A1298C polymorphism did not exhibit a similar association (21). These findings underscore the importance of personalized treatment and prevention strategies based on genotype.

Our results also indicate that the distribution of genotype and allele frequencies for the MTHFR and MTRR genes in our population aligns with findings from previous studies (20, 22). Notably, we observed that the high-risk group receiving folic acid supplementation had significantly elevated levels of PlGF, suggesting that folic acid may help prevent eclampsia by enhancing PlGF concentration in the body. By influencing the production of PlGF, folic acid may help regulate angiogenesis, promoting the formation of blood vessels in the placenta. This is essential for meeting the increasing demands for oxygen and nutrients during pregnancy, thereby reducing the risk of developing preeclampsia. This finding provides a promising avenue for future basic research and warrants further investigation.

Despite these advancements, our study has certain limitations that require consideration. Firstly, the sample size was not sufficiently large, necessitating further research with an expanded sample and multi-center studies. Secondly, while our findings suggest that folic acid may mitigate the risk of preeclampsia by influencing PlGF levels, we did not perform specific biochemical validations or mediation analyses in our current study. Future research should incorporate biochemical assays to measure PlGF and related markers in biological samples, allowing for a more comprehensive exploration of the underlying pathways. Thirdly, despite our comprehensive adjustments for confounding factors, unmeasured variables could still exist, potentially impacting our findings. Fourthly, the research sample predominantly consisted of the Han Chinese population, with limited data from other ethnic groups. Therefore, caution should be exercised when generalizing our conclusions to other countries and ethnicities.

In conclusion, based on genetic polymorphisms of MTHFR and MTRR, pregnant women who received appropriate folic acid supplementation exhibited higher levels of PlGF, demonstrating a significant association between folic acid and preeclampsia. Further evidence from randomized controlled trials and experimental studies is essential to clarify the exact nature of this interplay in the future.

## Data Availability

The raw data supporting the conclusions of this article will be made available by the authors, without undue reservation.
